# A Case of Ascending Colon Perivascular Epithelioid Cell Tumor Presenting with Intestinal Intussusception: Case Report

**DOI:** 10.70352/scrj.cr.24-0090

**Published:** 2025-06-17

**Authors:** Mitsuki Yokota, Hidekazu Takahashi, Hiromi Tsuji, Yuka Iwami, Juavi Jitjan Watsapol, Shohei Takaichi, Masakatsu Paku, Kazuya Iwamoto, Tomofumi Ohashi, Yujiro Nakahara, Tadafumi Asaoka, Chu Matsuda, Kazuhiro Nishikawa, Ichiro Takemasa, Takeshi Omori

**Affiliations:** 1 Department of Gastroenterology, Osaka Police Hospital, Osaka, Osaka, Japan; 2 Department of Pathology, Osaka General Medical Center, Osaka, Osaka, Japan

**Keywords:** perivascular epithelioid cell tumor (PEComa), colon, intussusception

## Abstract

**INTRODUCTION:**

Perivascular epithelioid cell tumors (PEComas) arising from the colon are uncommon. This case report describes a 40-year-old woman who presented with lower abdominal pain and was subsequently diagnosed with a colonic PEComa causing intestinal intussusception.

**CASE PRESENTATION:**

The patient initially presented with lower right abdominal pain. Computed tomography revealed an intestinal mass in the ileocecal region, prompting surgical intervention. Due to the nature of the mass, endoscopic repair was not feasible, and she underwent an emergency laparoscopic ileocecal resection. A significant mass was identified in the ascending colon, comprising proliferating spindle-shaped cells within the colonic wall. Immunohistological analysis revealed positive staining for smooth muscle actin (+), HMB-45 (+), and MelanA (±), confirming the diagnosis of PEComa. The patient recovered uneventfully and was discharged on postoperative day 7.

**CONCLUSIONS:**

Colonic PEComa is a rare malignancy. This case adds to the existing knowledge regarding intestinal intussusception caused by colonic PEComa.

## Abbreviations

AML
: angiomyolipomas
CCT
: clear cell tumors
CE
: contrast-enhanced
CT
: computed tomography
GIST
: gastrointestinal stromal tumor
HPF
: high power field
PEC
: perivascular epithelioid cells
PEComa
: perivascular epithelioid cell tumors
SMA
: smooth muscle actin

## INTRODUCTION

Perivascular epithelioid cell tumors (PEComas) are a rare group of stromal tumors derived from multipotent perivascular epithelioid cells (PEC). Colonic PEComas are particularly uncommon. This case report describes a patient with bowel obstruction caused by a rare colonic PEComa of the ascending colon. The patient underwent laparoscopic ileocecal resection for treatment. A literature review is also included.

## CASE PRESENTATION

A 40-year-old woman presented to our hospital with right lower abdominal pain. A contrast-enhanced (CE)-computed tomography (CT) scan showed an obstruction in the ileocecal region. However, against medical advice, the patient chose to leave the hospital.

The following day, her abdominal pain returned, and she was admitted to her previous hospital. An additional CT scan confirmed the bowel obstruction in the ileocecal region. The patient was then transferred to our hospital for surgery.

**Fig. 1:**
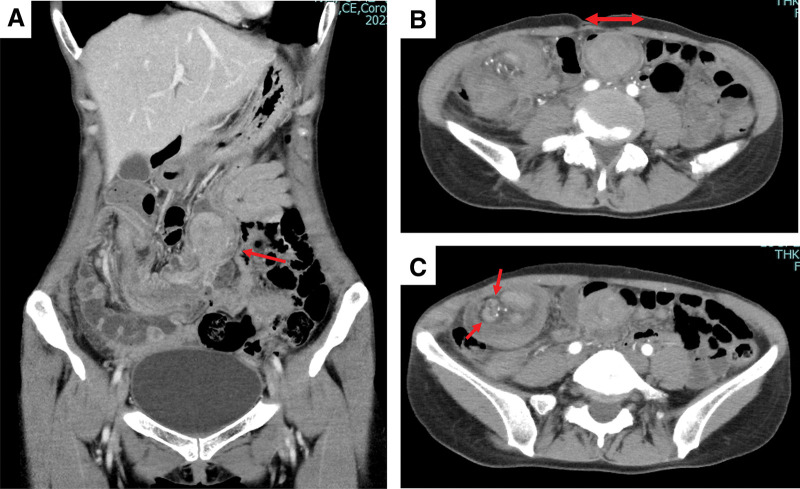
Contrast-enhanced CT scan of the abdomen (**A**) Superimposition of the ascending colon and the end of the ileum within the transverse colon. (**B**) 34 mm-sized mass in the advanced part of the colon. (**C**) Large intestinal peritoneal lymph nodes are observed.

Upon arrival at our hospital, the patient was alert and her vital signs were normal. However, she continued to experience right lower abdominal pain and guarding during palpation. A repeat CE-CT scan of the abdomen revealed a 34-mm mass located where the ascending colon meets the end of the ileum. The mass appeared to be compressing the transverse colon. An enlarged lymph node was also identified in the perigastric peritoneum near the mass (**Fig. 1**).

**Fig. 2:**
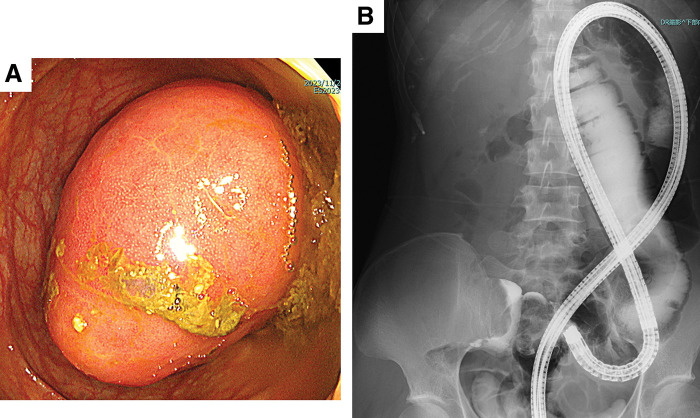
Colonoscopy and enema (**A**) erythematous submucosal tumour in the transverse colon. (**B**) Very short ascending colon with crab claw sign.

Endoscopy was initially attempted to see if the intussusception could be repaired without surgery. An endoscopic image (**Fig. 2A**) revealed a red mass in the transverse colon. The surface of the mass appeared to have normal mucosa lining, raising the suspicion of a tumor located in the submucosal layer. Fluoroscopy showed a marked shortening of the ascending colon with a crab claw sign (**Fig. 2B**).

**Fig. 3:**
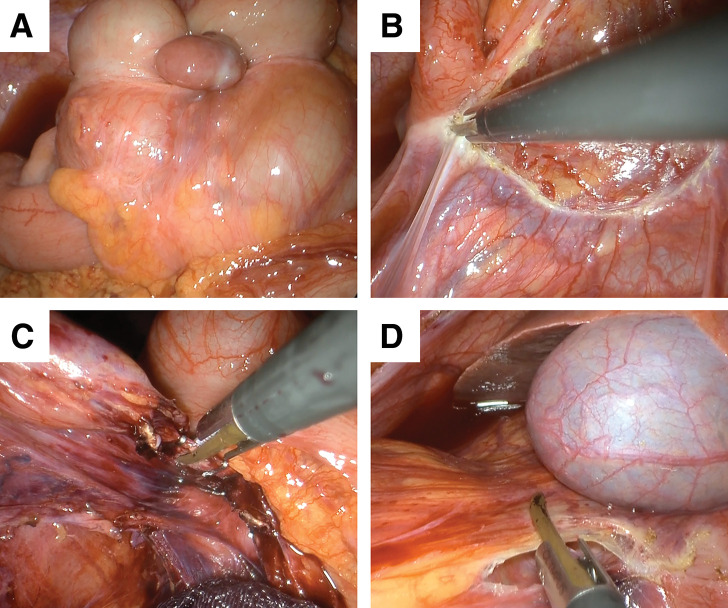
Surgical findings (**A**) Ileocecal stroma. (**B**) Ileocecal transfer. (**C**) ICA/ICV dissection. (**D**) Hepatic kyphosis transfer. ICA, ileocolic artery; ICV, ileocolic vein

Although endoscopic repair was attempted, it was technically challenging and ultimately unsuccessful. Based on endoscopic and CT findings, differential diagnoses included lipoma, gastrointestinal stromal tumor (GIST), and leiomyoma. Consequently, the patient underwent emergency laparoscopic surgery. The surgery was performed in the lithotomy position. A single-incision laparoscopic approach was used to perform an ileocecal resection with a D2 lymph node dissection (**Fig. 3**). This involved meticulous dissection of the ileocolic artery and vein, followed by mobilization of the right colon up to the hepatic flexure. The ileocecal region was then resected outside the abdomen (extracorporeal resection). Finally, a functional end-to-end anastomosis of the remaining small intestine and colon was created. The surgery was completed within 1 h and 52 min with minimal blood loss (approximately 0 mL).

**Fig. 4:**
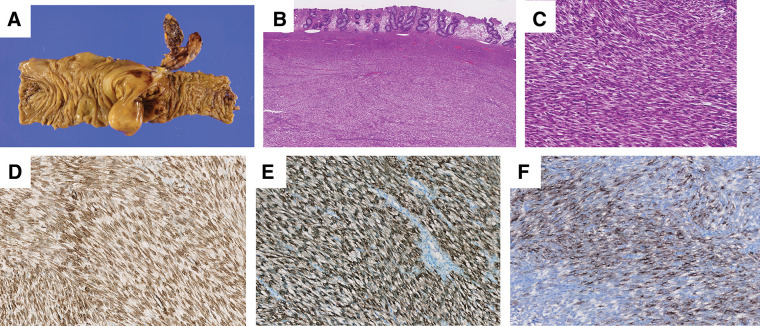
Histopathological and immunohistological examination (**A**) White, substantial mass with a maximum diameter of 61 mm was found in the ascending colon, with negative resection margins. (**B**) (Hematoxilyn Eosin stain, 40×) The mass was located in the submucosa and partly extended into the mucosal lining, intrinsic layer, intrinsic muscle layer and serosa. (**C**) (Hematoxilyn Eosin stain, 200×) Within the mass there were bundles of hyperplasia and convoluted spindled cells. (**D**) (SMA immunostaining 200×) Tumor is diffusely positive. (**E**) (HMB-45 immunostaining 200×) Tumor is diffusely positive. (**F**) (MelanA immunostaining 200×) Tumor is diffusely positive. SMA, smooth muscle actin

The resected specimen revealed a large white mass (61 mm diameter) located in the ascending colon with clear margins (**Fig. 4A**). The mass originated in the submucosa and partly invaded the mucosa, muscularis propria, and serosa (**Fig. 4B**). High power field (HPF) microscopic examination of the mass showed clustered and intertwining bundles of spindle-shaped cells (**Fig. 4C**). These cells did not exhibit significant atypia or pleomorphism. There was no evidence of coagulation necrosis or vascular invasion. The mitotic count was low (1/10 HPF). No metastasis was identified in the examined lymph nodes.

Immunohistochemical examination showed that the tumor cells were negative for CD34, S100, desmin, DOG-1, STAT-6, and epithlial membrane antigen (EMA). However, they were positive for smooth muscle actin (SMA) (**Fig. 4D**), HMB-45 (**Fig. 4E**), and Melan-A (weakly positive, **Fig. 4F**). The proliferation rate as measured by the Ki-67 labeling index was low (5% in hot spots). Based on the microscopic features and immunohistochemical pattern, the tumor was diagnosed as a PEComa.

The patient recovered smoothly and was discharged home on the 7th day after surgery.

## DISCUSSION

PEComas were first described by Bonetti et al. in 1992.^1)^ They observed that lung clear cell tumors (CCTs) and renal angiomyolipomas (AMLs) often shared a similar histological pattern with PEC.^2)^ This concept was expanded to encompass tumors with similar histology in various organs, including lymphangioleiomyomatosis, clear cell myelomelanocytic tumors, and certain unusual CCTs.^3,4)^ Notably, PEComas are distinguished by the prominence of epithelioid cells. The World Health Organization classification formally recognized PEComas as a distinct group of tumors arising from PECs.^5,6)^

Epidemiologically, PEComas are rare and predominantly affect middle-aged women (male-to-female ratio of 1:5, average age: 45 years).^7)^ The higher prevalence in women is suspected to be linked to female hormones, as most PEComas express progesterone receptors.^8)^ While PEComas can occur anywhere in the body, the most common locations are the kidneys, urogenital organs, and uterus.^9–12)^

PEComas are characterized by the proliferation of epithelioid or spindle-shaped cells with pale or pale acidophilic granulosa-like spherocytes arranged in a honeycomb pattern with intervening blood vessels.^13)^ Diagnosis relies heavily on immunostaining tests. These tests typically show positivity for markers associated with PECs, such as SMA, actin, and calponin, alongside markers for melanocytes like HMB45 and Melan-A.^14)^ In this specific case, the diagnosis of PEComa was confirmed by the presence of spindle-shaped cell proliferation and positive immunostaining for SMA, HBM45, and Melan A. It is still difficult to make a preoperative diagnosis of colonic PEComa based solely on imaging, and there are no clear reports on this so far. While there have been a case of PEComa in solid organs where preoperative diagnosis was possible, it was diagnosed after biopsy with immunohistochemical staining.^15)^ It is possible that a colonic PEComa could be diagnosed preoperatively through an endoscopic biopsy.

A PubMed search for “colon PEComa” covering the 10-year period from 2014 to 2024 identified six reported cases of PEComa arising within the colonic lumen.^16–21)^ These cases have been summarized in **Table 1**. The symptoms of colonic PEComa are diverse, but adult intussusception due to colonic PEComa is exceptionally rare, with only two documented cases reported in pediatric patients.^16,22)^ While intussusception itself is uncommon in adults, with an estimated 5% of all cases, the majority involve an underlying tumor.^23)^ Common culprits for bowel stalking in adults include colorectal cancer, polyps, Meckel’s diverticulum, colonic diverticulum, and strictures.^23,24)^ This case highlights the importance of recognizing colonic PEComa as a potential cause of adult intussusception alongside established etiologies.

**Table 1:** Colonic PEComa and symptoms

Publication year	Author	Age	Sex	Symptom	Tumor location
2020	Bennett et al.^16)^	67	Female	No symptoms	Ascending colon
2022	Razak et al.^17)^	30	Female	Abdominal pain	Cecum
2022	Kou et al.^18)^	12	Female	Abdominal pain, vomiting, Intussusception, Incarceration in the anus	Transverse colon
2022	Chua et al.^19)^	39	Female	Anal pain during defecation	Rectum
2023	Chen et al.^20)^	55	Female	Abdominal pain, abdominal mass	Ascending colon
2023	Yan et al.^21)^	Middle-aged	Female	No symptoms	Sigmoid colon

PEComas may be asymptomatic and found incidentally, or present with symptoms such as bleeding, abdominal pain, or intussusception.

PEComas are typically benign, although rare malignant forms exist.^25,26)^ Folpe et al. established a grading system to assess malignancy risk. This system identifies six high-risk factors: (1) tumor diameter of 5 cm or more, (2) invasive growth pattern, (3) severe nuclear atypia and increased cell density, (4) fission pattern (>1/50 HPF), (5) necrosis, and (6) vascular invasion.^8)^ The presence of only one high-risk factor classifies the tumor as having uncertain malignant potential. If two or more factors are present, the tumor is classified as malignant. Studies by Martignoni et al. suggest that malignant PEComas often originate in the kidney or uterus and can have a poor prognosis with various recurrence patterns, including hematogenous metastasis, lymph node metastasis, local recurrence, and peritoneal dissemination.^27)^ There are no specific reports indicating the exact malignancy rate, but Folpe et al. conducted a follow-up study on 26 cases of PEComa.^8)^ Their findings showed that, of the 24 cases tracked, 2 patients died due to PEComa, and 4 had metastasis or unresectable local lesions. Based on these results, the malignancy rate of PEComa might be around 25% as a surrogate marker.

Surgical resection is the mainstay treatment for the first occurrence of a resectable tumor; however, there is no standardized post-operative follow-up protocol.^25,28)^ In this case, the patient’s tumor fulfilled three high-risk factors, large diameter and high mitotic rate, invasive growth pattern. Therefore, a follow-up regimen similar to colorectal cancer guidelines was implemented, involving imaging studies every 6 months. Fortunately, the patient has remained recurrence-free for 6 months postoperatively.

## CONCLUSIONS

This case report describes a rare presentation of intussusception caused by a colonic PEComa, successfully treated with laparoscopic ileocecal resection. PEComas are typically benign, have malignant forms, and require close follow-up due to their aggressive potential. Given the rarity of colonic PEComa as a cause of intussusception, further case accumulation is necessary to establish optimal treatment strategies.

## DECLARATIONS

### Funding

The authors report no relationships relevant to the contents of this paper.

### Authors’ contributions

MY and HT were involved in the surgery.

MY collected the data and drafted the article.

HT helped revise the article.

All authors read and approved the final manuscript.

All authors accept responsibility for every aspect of this report.

### Availability of data and materials

Not applicable.

### Ethics approval and consent to participate

Not applicable.

### Consent for publication

Written informed consent was obtained from the patient for the publication of this report and the associated images.

### Competing interests

The authors declare that they have no competing interests.
